# The crossover design for migraine preventives: an analyses of four randomized placebo-controlled trials

**DOI:** 10.1186/s10194-019-1067-z

**Published:** 2019-12-27

**Authors:** Astrid Bjørke Jenssen, Lars Jacob Stovner, Erling Tronvik, Trond Sand, Grethe Helde, Gøril Bruvik Gravdahl, Knut Hagen

**Affiliations:** 1 Department of Neuromedicine and Movement Science, Norwegian University of Science and Technology, 7489 Trondheim, Norway; 2 Norwegian Advisory Unit on Headaches, St. Olavs University Hospital, Trondheim, Norway; 3 Department of Neurology and Clinical Neurophysiology, St. Olavs University Hospital, Trondheim, Norway; 4 Clinical Research Unit Central Norway, St. Olavs Hospital, Trondheim, Norway

**Keywords:** Headache, Preventive treatment, Carryover effect, Loss of follow-up

## Abstract

**Aims:**

To evaluate the crossover design in migraine preventive treatment trials by assessing dropout rate, and potential period and carryover effect in four placebo-controlled randomized controlled trials (RCTs).

**Methods:**

In order to increase statistical power, the study combined data from four different RCTs performed from 1998 to 2015 at St. Olavs Hospital, Norway. Among 264 randomized patients, 120 received placebo treatment before and 144 after active treatment.

**Results:**

Only 26 (10%) dropped out during the follow-up period of 30–48 weeks, the majority (*n* = 19) in the first 12 weeks. No period effect was found, since the treatment sequence did not influence the responder rate after placebo treatment, being respectively for migraine 30.5% vs. 27.4% (*p* = 0.59) and for headache 25.0% vs. 24.8% (*p* = 0.97, Chi-square test) when placebo occurred early or late. Furthermore, no carryover effect was identified, since the treatment sequence did not influence the treatment effect (difference between placebo and active treatment). There was no significant difference between those who received active treatment first and those who received placebo first with respect to change in number of days per 4 week of headache (− 0.9 vs. -1.3, *p* = 0.46) and migraine (− 1.2 vs. -0.9, *p* = 0.35, Student’s t-test).

**Conclusions:**

Summary data from four crossover trials evaluating preventive treatment in adult migraine showed that few dropped out after the first period. No period or carryover effect was found. RCT studies with crossover design can be recommended as an efficient and cost-saving way to evaluate potential new preventive medicines for migraine in adults.

## Introduction

Preventive medication is indicated for many migraine patients but used regularly by relatively few [1]. The randomized controlled trial (RCT) is the gold standard for evaluating the effect of preventive treatment in patients with migraine. According to the current guidelines for RCTs of preventive treatment in migraine published in 2012, either a parallel or crossover study design can be used, depending on the trial’s objectives [2].

The guidelines also recommend that a placebo arm always should be included in trials [2], but this has been done in less than 10% of RCTs evaluating multiple preventive drugs [3]. Summaries of results of RCTs on oral medication for migraine prevention including placebo arms have shown that 21–22% of patients had at least 50% reduction of attacks during oral placebo treatment [4, 5]. Higher responder rates have been found in studies with parallel group design compared with crossover studies [4], possibly because of higher positive expectations in participants in parallel studies than in crossover studies [4].

In contrast to the guidelines for RCTs of drug treatment in migraine, recently published guidelines for controlled trials of preventive treatment recommend parallel-group design over crossover design [6, 7]. The main argument against the crossover design is the possibility of carryover effect even when a washout period is being used. Furthermore, because of the long study period there may be high withdrawal rates and increase in protocol deviations, and marked spontaneous fluctuations in disease activity over time, known as period effects, may occur. The scientific documentation for these arguments is modest. It should be highlighted that the listed reference for these arguments, appearing in the guidelines for chronic migraine [6], was published in 1987 [8], almost 20 years before the diagnosis of chronic migraine was defined [9]. Thus, analytic studies are needed to assess the value of the crossover design for RCTs in migraine preventive medication.

It has been argued that potential carry-over effects will be hard to detect in a single study, in part because the studies are powered to detect a relatively large and clinically meaningful effect. Carry-over effects are likely to be smaller, and the statistical power is much smaller since only half the participants receive the active medication before placebo [10]. Similar arguments can be made about the period effect.

During a period of 20 years, four methodologically very similar randomized placebo-controlled trials in migraine using a crossover design have been performed at St. Olavs University Hospital in Trondheim, Norway [11–14]. The aim of the present study was to evaluate the crossover design for trials on episodic migraine preventive medication by combining the data from these four studies, in this way achieving higher statistical power for assessing dropout rate and potential period and carryover effects.

## Methods

### Study design

Data included in this study was collected from four placebo-controlled RCTs conducted between 1998 and 2015 at the Department of Neurology at St. Olavs University Hospital in Trondheim, Norway [11–14]. All studies included migraine patients with at least two migraine attacks during the last 4 weeks and had a crossover design. In all studies participants kept a headache diary throughout the study and had regular consultations with the study neurologist, and repeated telephone calls by a study nurse. The first three studies evaluated antihypertensive medication (candesartan, lisinopril and propranolol versus candesartan) [11–14], whereas the last published study evaluated a dietary supplement, acetyl-l-carnitine (ALCAR) [14]. In all four studies participants received maximum number of tables in week 2–11, and half dose in week 1 and 12.

Study details that differ between the studies are summarized in Table 1. One of the studies had a double crossover design, i.e. comparing candesartan with an active drug (propranolol) in addition to placebo. In the three studies using antihypertensives as active substance [11–13], there was a significant effect of the active substance over placebo. This was not shown in the study using ALCAR as active substance [14]. One important difference between the studies was that placebo tablets were used in the baseline period (“placebo-run-in”) and washout period in the two first studies [11, 12], but not in the two last ones [13, 14]. Having a placebo run-in was recommended in the first and second edition of guidelines for controlled trials of drugs in migraine in order to exclude placebo responders [15, 16], but not in the third edition [2].

**Table 1:** Summary of study details which differ between studies

Authors	Schrader et al. [11]	Tronvik et al. [12]	Stovner et al. [13]	Hagen et al. [14]
Publication year	2001	2003	2014	2015
Number included	60^1^	60^2^	72	72
Age range at inclusion	19–59	18–65	18–65	18–65
Number of migraine attacks per month at inclusion	2–6	2–6	≥2	≥2
Number of treatment periods	2	2	3	2
Duration of follow-up, weeks	30	32	48	32
Active substance(s)	Lisinopril	Candesartan	Candesartan and Propanolol^3^	Acetyl-L-carnitine
Effect of active versus placebo	Yes	Yes	Yes	No
Blinding	Double	Double	Triple^4^	Triple^4^
Placebo tablets in baseline period	Yes	Yes	No	No
Placebo tablets in washout period	Yes	Yes	No	No

^1^63 entered baseline period, but 3 were not randomized after run-in period because of < 2 attacks/month (*n* = 2) or for no specific reason (*n* = 1)

^2^75 entered baseline period, but 15 were excluded because of < 2 attacks/month (*n* = 5), > 6 attack/month (*n* = 7) or for other reasons (*n* = 3)

^3^This study was a double crossover study

^4^Meaning that the statisticians were also blinded

In the double crossover study, there were three treatment periods [13]. Twenty-four patients (intention-to-treat-analysis) got placebo first, 16 got placebo after treatment with one active substance (either candesartan or propranolol), and 21 got placebo after both active substances. Furthermore, two of the studies were run as double blind, and two as triple blind, i.e. not only were participants and study personal blinded, but also the statistician [13, 14].

The primary efficacy variables in the two first studies were headache days and migraine days per 4 weeks [11, 12], whereas the primary efficacy variables in two last studies was number of days with moderate to severe headache lasting ≥4 h or being treated with attack medication [13, 14]. To make data uniform regarding headache days and migraine, we had to perform a new data entry of all headache diaries for three studies [12–14] (*n* = 204). In the headache diary the participants had to register all headaches. A migraine day was defined as a day with headache the patients themselves considered to be migraine. Number of headache days/month was the sum of migraine days and days with non-migrainous headache. Responders were defined as patients with at least 50% reduction in headache days or migraine days in the 12-week period compared to baseline. For individuals with incomplete headache diaries, the last-observation-carried-forward strategy was used.

### Ethics

The present study (2017/2050/REK Midt) and all the four original studies [11–14] were approved by the Regional Committee for Ethics in Medical Research, Norway. The two last studies [13, 14] were also approved by the Norwegian Medicines Agency, but this was not needed at the time the two first studies were performed. All participants had signed a written consent declaration in the original studies.

### Statistical analysis

Differences between proportions were analyzed by Chi-squared test including evaluation of dropouts and responder rates.

Period effect (i.e. spontaneous fluctuations in disease activity) was evaluated by comparing the mean number of headache days/migraine days (using Wilcoxon’s paired signed rank test) in the 12 weeks on placebo related to whether the placebo period occurred early or late after the patient was included in the study. Furthermore, the period effect was also evaluated by analyzing the placebo response by comparing responder rate for participants who received placebo treatment in the first period compared to those who received placebo after active treatment. To minimize the influence of dropouts, the responder rate in each period was estimated based on eligible participants (number included minus dropouts). The placebo response was also evaluated by comparing days with headache and migraine during baseline with the 12 weeks of placebo-treatment using Wilcoxon’s paired signed rank test.

Because placebo tablets were used in the baseline period in the two first studies [11, 12], we presented separate data for studies with [11, 12] and without placebo [13, 14] in the baseline period, as well as merged data from all four studies.

There are no established rules or guidelines on how to calculate potential carryover effect, defined as a prolonged effect of active treatment that persist into the subsequent period [17]. However, if such effect exists, one should expect that treatment sequence, i.e. placebo before active substance or vice versa, would influence the efficacy. By using Student’s t-test we evaluated difference in number of days per 4 weeks of headache and migraine between active treatment and placebo according to sequence. In the analyses of carryover effect, we excluded participants included in the last performed study because no effect of active treatment (ALCAR) compared to placebo treatment was found [14]. Another indirect way to evaluate carryover effect was to compare responder rate related to treatment sequence as mentioned above. Finally, the mean number of headache/migraine days in the washout period was compared in relation to treatment sequence, as this period would presumably be even more affected by potential carryover effect.

The SPSS statistical program (version 25) was used for the statistical analyses and two-sided *p* values less than 0.05 were regarded as statistically significant.

## Results

In the two first studies, a total of 138 migraine patients entered the baseline period using placebo tablets, whereas in the two last studies 164 migraine patients entered the baseline period without using placebo tablets. The proportion of patients that had to leave the study because of too few migraine attacks was not different among those who did and did not use placebo tablets during the baseline period (5.8% versus 6.7%, *p* = 0.82).

Baseline characteristics of the 264 randomized individuals in the four studies are summarized in Table 2.

**Table 2:** Background variables in the different studies

Author	Schrader et al. [11]	Tronvik et al. [12]	Stovner et al. [13]	Hagen et al. [14]	All
Publication year	2001	2003	2014	2015	–
Number included	60	60	72	72	264
Mean age (SD)	41.1 (10.2)	42.9 (12.0)	37.3 (10.7)	38.9 (12.3)	39.9 (11.5)
Female sex, n (%)	50 (83.3)	47 (78.3)	59 (81.9)	63 (87.5)	219 (83.0)
Migraine with aura, n (%)	27 (45.0)	28 (46.7)	33 (45.8)	23 (31.9)	111 (42.0)
Mean BMI (SD)	24.3 (4.4)	25.3 (4.3)	NA^1^	24.4 (3.7)	24.7 (4.1)
Mean age at first migraine attack (SD)	17.1 (7.7)	19.6 (9.1)	17.9 (10.7)	20.1 (9.3)	18.7 (9.3)
Mean self-reported frequency of migraine attacks/months (SD)	4.3 (1.6)	3.7 (1.2)	4.8 (3.6)	3.6 (1.6)	4.1 (2.3)
Mean migraine days/4 weeks (SD)	6.6 (3.4)	5.7 (2.9)	5.3 (3.1)	5.3 (2.3)	5.7 (2.9)
Mean headache days/4 weeks (SD)	9.7 (5.2)	8.4 (3.9)	8.2 (4.3)	6.4 (2.8)	8.1 (4.2)

^1^Not available

### Dropout rates and per protocol completers

After randomization, 26 out of 264 (9.8%) dropped out during the follow-up period, the majority (*n* = 19) early in the first 12-week period. During the first period higher dropout rate was found among participants who were randomized to active treatment (16 out of 144) than among those who got placebo treatment (3 out of 120) (11.1% versus 2.5%, *p* = 0.008). Overall, 208 (79%) completed the studies per protocol.

### Period effect: placebo response related to treatment sequence

At baseline mean number of headache days tended to be somewhat lower for those who got placebo treatment first (*n* = 120) than for those who got placebo after active treatment (*n* = 144) (overall 7.6 vs. 8.5 days, *p* = 0.08), whereas migraine days at baseline were nearly identical (5.8 vs. 5.6 days, *p* = 0.68) (Table 3). No significant difference in responder rate was found for individuals who received placebo in the first period compared to those who got placebo after active treatment, the proportions for all four studies being respectively 27.4% vs. 30.5% (*p* = 0.59) for migraine days and 24.8% vs. 25.0% (*p* = 0.97) for headache days (Table 3). For those who got placebo-treatment in the first period, the number of days per 4 weeks decreased with a mean of 1.4 for migraine (5.8 in run-in versus 4.4, *p* < 0.001) and 1.7 for headache (7.6 run-in versus 5.9, *p* < 0.001). Correspondingly, for those who got placebo after active treatment the number of days per 4 weeks decreased with a mean of 1.0 for migraine (5.6 in run-in versus 4.6, *p* < 0.001) and 2.1 for headache (8.5 run-in versus 6.4, *p* < 0.001) (Table 3). There was no significant difference when comparing the reduction in migraine days during placebo treatment from baseline period related to treatment sequence (*p* = 0.87).

**Table 3:** Number of dropouts and days with headache and migraine per 4 weeks related to treatment sequence and use of placebo in baseline period (*N* = 264)

	Placebo given in baseline period		Placebo not given in baseline period		All	
Treatment sequence	Placebo in first period	Active treatment in first period	Placebo in first period	Active treatment in first period	Placebo in first period	Active treatment in first period
Number included	60	60	60	84	120	144
Dropouts during first period	2	5	1	11	3	16
Migraine days/month, mean (SD)						
Baseline period	6.0 (2.9)	6.2 (3.4)	5.5 (2.4)	5.2 (2.9)	5.8 (2.7)	5.6 (3.1)
Placebo treatment period	5.0 (3.0)	5.5 (3.2)	3.9 (2.2)	3.9 (2.7)	4.4 (2.6)	4.6 (3.0)
Washout period	4.6 (3.9)^1^	5.9 (4.3)^1^	3.8 (3.1)^2^	4.0 (2.7)^2^	4.2 (3.5)^2^	4.8 (3.6)^2^
Number of responders (% of eligible)^4^	16 (27.6)^5^	15 (27.3)^5^	16 (27.1)^5^	24 (32.9)^5^	32 (27.4)^5^	39 (30.5)^5^
Headache days/month, mean (SD)						
Baseline period	8.4 (3.7)	9.8 (5.3)	6.8 (3.0)	7.7 (4.1)	7.6 (3.5)	8.5 (4.8)
Placebo treatment period	7.0 (4.1)	7.4 (4.1)	4.9 (2.8)	5.7 (3.9)	5.9 (3.6)	6.4 (4.1)
Washout period	6.1 (4.3)^3^	9.1 (6.5)^3^	4.5 (3.5)^1^	5.6 (4.2)^1^	5.3 (4.0)^3^	7.1 (5.6)^3^
Number of responders (% of eligible)^4^	13 (22.4)^5^	13 (23.6)^5^	16 (27.1)^5^	19 (26.0)^5^	29 (24.8)^5^	32 (25.0)^5^

Washout headache/migraine according to treatment sequence compared by Students t-test: ^1^*P* ≥ 0.08 ^2^*P* ≥ 0.16 ^3^*P* ≤ .005

^4^ Responders are defined as having at least 50% reduction in number of days/month. Eligible is defined as number included minus dropouts

Number of responders related to treatment sequence compared by Chi-square test: ^5^*P* ≥ 0.47

### Analyses of carryover effect

**Fig. 1:**
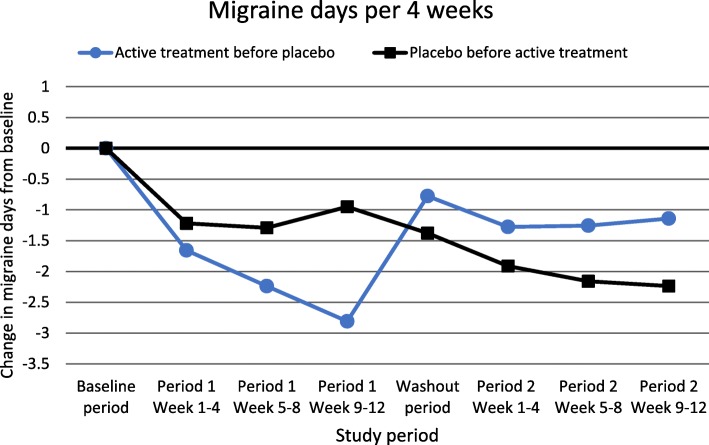
Migraine days per 4 weeks in relation to treatment sequence (square: Placebo first, *n* = 80; circle: Active treatment first, *n* = 96). Data from the study published in 2015 (the ALCAR-study) were omitted from this analysis due to lack of efficacy vs. placebo

**Fig. 2:**
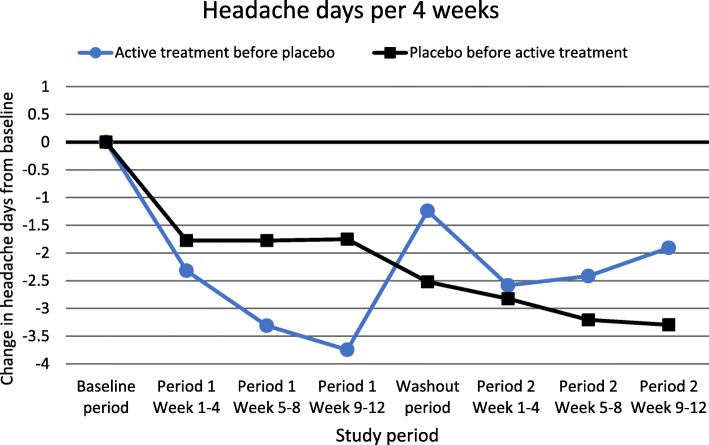
Headache days per 4 weeks in relation to treatment sequence (square: Placebo first, *n* = 80; circle: Active treatment first, *n* = 96). Data from the study published in 2015 (the ALCAR-study) were omitted from this analysis due to lack of efficacy vs. placebo

Days per 4 weeks during follow-up according to treatment sequence are presented in Fig. 1 for migraine and Fig. 2 for headache. As demonstrated, a marked worsening of days with migraine and headache occurred in the washout period for those who got active treatment in the first period, whereas no such worsening in the washout period was seen among those who received placebo in the first period. The number of headache days in the washout period was significantly higher after active treatment than after placebo treatment (7.9 days vs. 5.8 days, *p* = 0.008) (Fig. 2).

In the statistical analyses of potential carryover effect, we found that the order of treatment did not influence the efficacy of active treatment versus to the placebo period (Table 4). No significant difference in change of days per 4 weeks of migraine (*p* = 0.35) or headache (*p* = 0.46) was found when comparing treatment sequence (active or placebo first) (Table 4).

**Table 4:** Analyses of carry-over effect: effect of active treatment compared to placebo related to treatment sequence (*N* = 176)^1^

Order of treatment	Number with complete data	Change in migraine days/4 weeks, mean (95% CI)	Change in headache days/4 weeks, mean (95% CI)
Placebo before active treatment	80	−0.90 (−1.39, −0.40)	−1.26 (− 1.95, − 0.57)
Placebo after active treatment	96	− 1.22 (− 1.69, − 0.74)	−0.90 (− 1.58, − 0.23)
P-value^2^		0.35	0.46

^1^2001, 2003 and 2014 data included

^2^Evaluated by Student’s t-test

## Discussion

Using data from four crossover randomized trials evaluating preventive treatment in migraine, we found that the response to placebo treatment was consistent during follow-up, regardless of whether it came early or late in the study. Very few dropped out after the first period and no carryover effect was identified.

Crossover studies are not recommended in current guidelines for controlled trials of preventive treatment of chronic migraine owing to concerns over potential carryover effect, challenges with withdrawals, and fluctuation in treatment effect over time [6]. However, there is little documentation that these concerns are warranted, and the present study does, in our opinion, challenge these arguments. To the best of our knowledge, absence of carryover or period effect has not previously been clearly demonstrated in other cross-over studies evaluating migraine or other types of headache.

### Dropout rates

A 9.8% dropout rate was observed during the 30–48 weeks follow-up, the majority early in the first 12-week period. Overall, 79% of the participants completed the study per protocol. In comparison, a dropout rate of 4% was reported in a 22-week follow-up in a Norwegian placebo-controlled randomized crossover study of episodic migraine evaluating treatment with melatonin [18]. Moreover, in three crossover studies comparing two different active drug, dropout rates between 0% and 10% % were found [19–21]. In more recently published large-scale RCTs with parallel group design, the proportion of dropouts have varied between 5.4–8.5% during 12 weeks’ treatment with calcitonin gene-related peptide (CGRP) antibodies [22–26], 8.5–13% during 24 weeks’ treatment with onabotulinumtoxin A [27, 28], and 31–46% during 26 weeks’ treatment with topiramate [29–31]. Thus, the dropout rates in our studies with crossover design were not substantially higher than those in comparable and relatively recent parallel group studies [32]. The impression is confirmed by looking at a meta-analysis including more than 100 RCTs on migraine prevention from 1970. In this study mean dropout rates were almost identical for studies with crossover (21.2%, SD 14.2, *n* = 59 studies) and parallel group design (21.1%, SD 14.0, *n* = 55 studies) [32].

### Fluctuation in treatment effect over time

The proportion of placebo responders was consistent during the follow-up period, being respectively 27–31% for migraine and 25–27% for headache. In comparison, previous meta-analyses have reported placebo responder rates in migraine preventives studies of respectively 21% (95% CI 13–28%) based on pooled data of 17 studies [4] and 22% (95% CI 17–28%) based on 26 studies [5]. The responder rate of active treatment in the present study was 47% for headache and 49% for migraine, somewhat higher than the mean of 26 studies showing a responder rate of migraine of 41% (95% CI 37–45%) [5]. One can argue that the consistent placebo-effect during the follow-up reported in our study, may be related to type of migraine patients included in these single center studies. In our opinion this explanation is less likely, but replication of the present results is needed.

### The possibility of a carryover effect

A prolonged effect of active treatment that persists into the subsequent placebo period is called the carryover effect. If a long-lasting carryover effect had been present, one should have expected an extended period without worsening of migraine and headache, which is not seen (Figs. 1 and 2). It should be mentioned that the half-lives of candesartan, lisinopril and propranolol are very short (12 h or less), and most of the active drug will be eliminated during very few days [33–35]. Thus, most likely a potential long-lasting carryover effect would have to be caused by other mechanisms than a direct effect of the drug itself. However, in the present study we did not find evidence of a carryover effect. Firstly, the order of treatment did not influence the efficacy of active treatment versus placebo measured by change in number of days with headache and migraine per 4 weeks. Secondly, patients experienced a marked worsening when they entered the washout period after the active treatment period, whereas no such worsening was seen when placebo was given in the first period (Fig. 1). In fact, the number of headache days in the washout period was significantly higher after active treatment than after placebo treatment. Based on the present study, a washout-period of 4 weeks seems sufficient to eliminate the possibility of a carryover effect of candesartan, lisinopril and propranolol, and probably other drugs with short half-lives.

### Impact of placebo tablets in baseline and washout

Placebo tablets was used in baseline period and washout period in the two first studies. The main reason for such strategy was to eliminate prominent “placebo responders”. There was, however, no difference in number of patients who had to be excluded from the study, owing to too few attacks depending on whether placebo was used during baseline or not. Furthermore, also in the washout period the use of placebo tablets did not have evident impact, because the increase in days with migraine and headache in the washout period after active treatment was even more marked when placebo tablets were given than when no tablets were given.

### Strengths and study limitation

The number of withdrawals was low and proportion of completers high, probably caused by close follow-up by the study nurse. The design of the four studies was nearly similar, but not identical. We were able to investigate the influence of study differences by performing separate analyses for studies with and without use of placebo tablets in baseline and washout period. Furthermore, in the analyses of carryover effect, we excluded the study evaluating ALCAR because no effect of active treatment was found compared to placebo. However, in order to preserve the advantages by combined data, we did not separate regarding other (probably less important) methodological differences e.g. duration of follow-up. The present study only included drugs with short half-lives. Thus, we cannot rule out the possibility of a prominent carryover effect for drugs with much longer half-lives. The present study included 264 migraine patients in four single center RCTs, three trials evaluating antihypertensives and one ALCAR. However, it should be mentioned that generalization of the present results should be done with caution, because all studies were performed in one center, there were only four RCTs, and only three types of drugs were analyzed. The inclusion period of the four studies varied between 1.3–3 years [11–14]. Thus, because patients were included throughout the year, the potential effect of seasonal variation of migraine attacks was minimized.

### Crossover design: pro and cons

The main advantage of the crossover design is that by allowing paired statistics it has much greater power than the parallel-group design, hence needing a much lower sample size. The crossover design is approximately eight times more powerful than parallel studies [16, 36]. Hence, in the present four crossover studies between 60 and 72 patients were randomized [11–14]. In contrast, the corresponding number of randomized patients included in the parallel studies with CGRP antibodies have varied between 410 and 1130. This means that evaluation of new and promising preventive medicines in migraine, at least those with a short half-life, can be performed by single centers with RCT with a crossover design. Based on the present analyses, neither high dropout rate, carryover effect, or period effect seem to be valid arguments against RCT with crossover design in migraine prevention. This was true even for the double crossover study where participants were followed for nearly 1 year [13]. Interestingly, a gradually increasing effect of active medication is seen over the three months (Figs. 1 and 2). Thus, considering the low dropout rate in these studies, we suggest that longer treatment periods than 12 weeks could be considered in order to capture more long-term effects of preventive treatments.”

## Conclusions

By analyzing data from 264 adult migraine patients included in four crossover randomized trials evaluating preventive treatment, we found no period effect, no carryover effect and low dropout rates. For single centers, wishing to evaluate promising new preventive medication in adult migraine, we recommend RCTs with crossover design, at least for the early trials. Because treatment seemed to be most effective in the last 9–12 weeks, treatment periods longer than12 weeks can be considered.

## Data Availability

Part of the dataset supporting the conclusions of this article is available on request to the corresponding author.

## Abbreviations

ALCAR: Acetyl-l-carnitine
CGRP: Calcitonin gene-related peptide
RCT: Randomized controlled trials
SD: Standard deviation
